# The “Penumbra Sign” on Magnetic Resonance Images of Brodie’s Abscess: A Case Report

**DOI:** 10.5812/iranjradiol.4493

**Published:** 2011-12-25

**Authors:** Ahmadreza Afshar, Afshin Mohammadi

**Affiliations:** 1 Department of Orthopedics and Hand Surgery, Imam Khomeini Hospital, Urmia University of Medical Sciences, Urmia, Iran; 2 Department of Diagnostic Radiology, Imam Khomeini Hospital, Urmia University of Medical Sciences, Urmia, Iran

**Keywords:** Abscess, Osteomyelitis, Magnetic Resonance Imaging

## Abstract

This report presents the “penumbra sign” of a Brodie’s abscess in a 69-year-old male patient. The lesion was located in the proximal metaphysis of the left tibia. Histopathology confirmed the diagnosis of subacute osteomyelitis. The penumbra sign on magnetic resonance (MR) images is a helpful sign for the diagnosis of Brodie’s abscess.

## 1. Introduction

Brodie’s abscess is a bone abscess described as a localized primary purulent collection and a sclerotic wall. Brodie’s abscess is usually confined to the metaphysis [1]. There may be ambiguity in differentiating Brodie’s abscess from other malignant and benign osseous lesions radiologically [2]. It has been showed that the “penumbra sign” on magnetic resonance (MR) imaging is useful for discriminating subacute osteomyelitis from other bone lesions [2][3]. The penumbra sign is a rim lining of an abscess cavity with higher signal intensity than that of the main abscess on T1-weighted images. This report presents the “penumbra sign” on MR images of a Brodie’s abscess in the proximal metaphysis of the left tibia. We present this case of Brodie’s abscess on magnetic resonance imaging (MRI) with “penumbra sign” differentiating it from malignant bone tumors.

## 2. Case Presentation

The patient was a 69-year-old male retired military officer. His chief complaint was left knee pain. He had no systemic illness. On examination, he had crepitation and mild joint effusion in the left knee. The left knee had a normal range of motion. There was a surgical scar on the medial side of his left proximal leg. He had a parachute accident at age 33 causing trauma to his left knee. He remembered that he was told about developing a bone infection at his proximal tibia and had undergone surgery then. After being discharged, he was not given any medication and was not told about any further necessary treatment in the future. He returned to his service and continued his normal activities. During the following years he did not have any symptoms at his previously injured site and no systemic illness.

At the time of examination, there was no sign of acute or chronic infection (swelling, tenderness, redness, sinus and discharge) around the left knee. Erythrocyte sedimentation rate (ESR), C-reactive protein and leukocyte count were normal.

**Figure 1:**
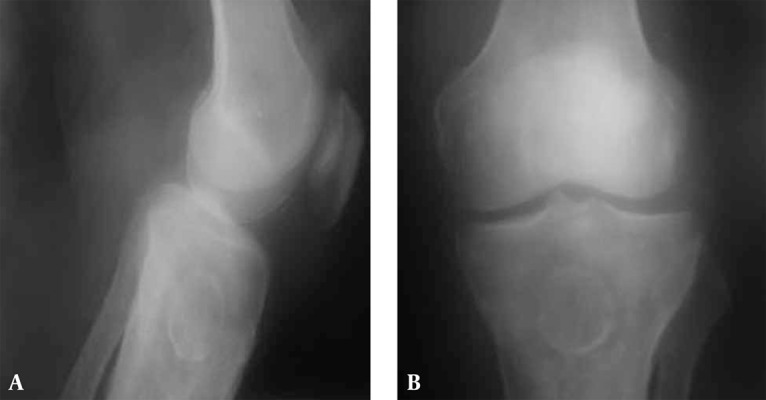
A-69-year-old male with left knee pain and a well corticated ossified intramedullary lesion in the proximal tibial metaphysis; A, Lateral view; B, Antero-posterior view

Plain roentgenograms of the left knee (Figure 1A and B) showed a well-defined and well-corticated ossified intramedullary lesion in the proximal tibial metaphysis. The lesion was accompanied by perilesional ill-defined osteoporotic changes and an area of hyperdense bone within the osteoporotic area was seen. Irregular thick perilesional periosteal reaction at the tibial methaphysis was also seen.

**Figure 2:**
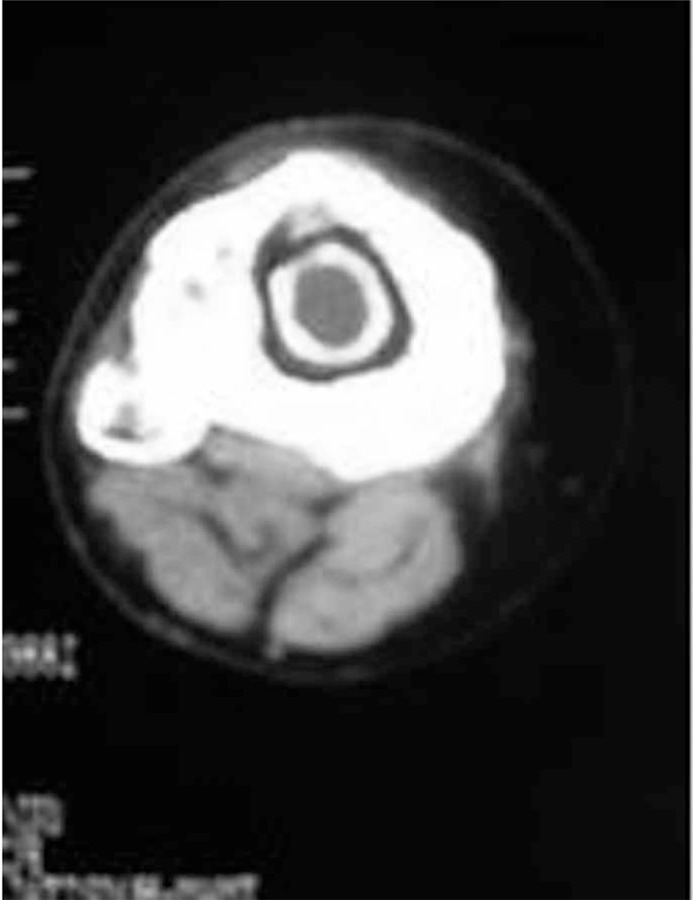
CT scan of the proximal left leg in the same patient shows a central intramedullary hypodense cystic lesion with a thick rim of ossification.

Proximal left leg Computed Tomography (CT) scan without contrast (Figure 2) showed evidence of a central intramedullary hypodense cystic lesion with thick rim ossification in the proximal tibial methaphysis. Extensive thick well-circumscribed periosteal reaction and bone sclerosis around the lesion in the proximal tibial methaphysis was also detected. No evidence of soft tissue lesion or cortical destruction around the lesion was seen.

**Figure 3:**
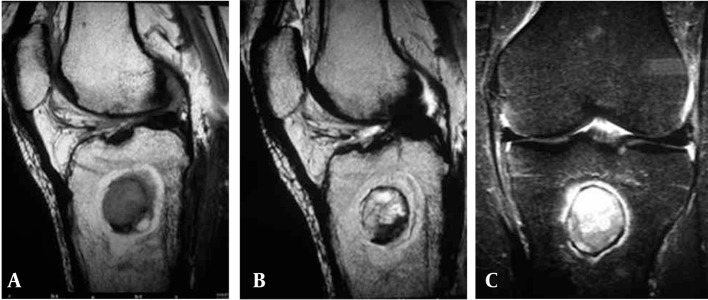
MRI of the same patient; A, T1-weighted TSE sequence reveals the penumbra sign; B, T2-weighted TSE sequence reveals the penumbra sign; C, MR image STIR-TSE sequence shows the penumbra sign.

Left knee MRI demonstrated the penumbra sign. The MRI showed a well-defined central intramedullary cystic lesion in the proximal tibial metaphysis. The central part of the lesion was hypointense on T1-weighted images (Figure 3A) and hyperintense on T2-weighted images (Figure 3B) and short tau inversion recovery (STIR)-TSE sequence (Figure 3C) that showed this part has high viscosity content. A thin layer of signal void lesion around the cystic central part in different sequences due to bone formation and sclerosis was seen. At T1 sequences a thick rim of discrete peripheral zone of higher signal intensity than the central bony abscess cavity due to highly vascularized granulation tissue was seen. Hyperintensity of the outer layer indicates that this layer is not due to bone edema alone. Thick layer perilesional bone formation and sclerosis as a hyposignal on T1, T2 and STIR sequences were seen. Joint effusion in the suprapatellar bursa was also detected.

The patient underwent surgical drainage of the abscess cavity and reconstruction of the lesion site. Histopathology confirmed the diagnosis of subacute osteomyelitis (Brodie’s abscess). The patient is in good general condition and there has been no sign of recurrence of local complication as cutaneous fistula in a 2-year-follow up.

## 3. Discussion

The penumbra is defined as partial or lighter shadow of an eclipse. It refers to the thin layer of granulation tissue that lines the abscess cavity in subacute osteomyelitis [3][4].

Marti-Bonmati et al. are credited for the first description of the “target” appearance of Brodie’s abscess on MRI; a center, two rings and a peripheral halo [5]. The “penumbra sign” is comprised of four sections; namely, a central core which represents the abscess cavity is composed of a high protein component and appears as low signal intensity on T1-weighted and high on T2-weighted and STIR images; the first layer is isointense to the muscle which is composed of a granulation layer. The second layer is hypointense on all sequences due to reactive new bone formation caused by chronic inflammation and an outer layer which is a peripheral halo of low signal intensity ring due to edema on T1-weighted images [4].

The “penumbra sign” is not pathognomonic but a characteristic MRI feature of subacute osteomyelitis [3][4][5][6]. It has been reported in cases of eosinophilic granuloma, chondrosarcoma, benign cystic lesions of the bone and intraosseous ganglion [4].

This sign on MRI has a sensitivity of 75% and specificity, positive and negative predictive values all higher than 90% in the diagnosis of subacute osteomyelitis [3][4][7].

McGuinness B et al. reported that the penumbra sign has a high specificity of 96% and a low sensitivity of 27% for diagnosis of musculoskeletal infection [8].

Radiologically, there is obscurity in distinguishing subacute osteomyelitis from other malignant and benign bone lesions. The radiologic manifestation of our case was similar to the previous cases [6][8]. However, the “penumbra sign” on MRI is helpful in distinguishing between subacute osteomyelitis from other osseous lesions [3].

## References

[1] Kanoun ML, Khorbi A, Khmiri C, Tebourbi A, Hadded N, Boughzala W (2007). [Diagnosis and treatment of Brodie’s abcess in adults: about twenty cases].. Tunis Med.

[2] Shih HN, Shih LY, Wong YC (2005). Diagnosis and treatment of subacute osteomyelitis. J Trauma.

[3] Grey AC, Davies AM, Mangham DC, Grimer RJ, Ritchie DA (1998). The ‘penumbra sign’ on T1-weighted MR imaging in subacute osteomyelitis: frequency, cause and significance. Clin Radiol.

[4] Davies AM, Grimer R (2005). The penumbra sign in subacute osteomyelitis.. Eur Radiol.

[5] Marti-Bonmati L, Aparisi F, Poyatos C, Vilar J (1993). Brodie abscess: MR imaging appearance in 10 patients.. J Magn Reson Imaging.

[6] Marui T, Yamamoto T, Akisue T, Nakatani T, Hitora T, Nagira K (2002). Subacute osteomyelitis of long bones: diagnostic usefulness of the “penumbra sign” on MRI.. Clin Imaging.

[7] Shimose S, Sugita T, Kubo T, Matsuo T, Nobuto H, Ochi M (2008). Differ ential diagnosis between osteomyelitis and bone tumors.. ActaRadiol.

[8] McGuinness B, Wilson N, Doyle AJ (2007). The “penumbra sign” on T1-weighted MRI for differentiating musculoskeletal infection from tumour.. Skeletal Radiol.

